# Atelectasis and lung changes in preterm neonates in the neonatal period: a blind radiological report and clinical findings

**DOI:** 10.5935/0103-507X.20190047

**Published:** 2019

**Authors:** Anne Karoline Santos, Jaqueline Silveira, Valéria Cabral Neves, Talita Gianello Gnoato Zotz, Arlete Ana Motter, Marimar Goretti Andreazza

**Affiliations:** 1 Programa de Atenção Hospitalar em Saúde da Criança e Adolescente, Complexo Hospital de Clínicas, Universidade Federal do Paraná - Curitiba (PR), Brasil.; 2 Unidade de Terapia Intensiva Pediátrica, Complexo Hospital de Clínicas, Universidade Federal do Paraná - Curitiba (PR), Brasil.; 3 Curso de Fisioterapia, Universidade Federal do Paraná - Curitiba (PR), Brasil.; 4 Unidade de Terapia Intensiva Neonatal, Complexo Hospital de Clínicas, Universidade Federal do Paraná - Curitiba (PR), Brasil.

**Keywords:** Infant, premature, Thorax/diagnostic imaging, Pulmonary atelectasis, Diagnostic imaging, Premature birth, Intensive care units, neonatal

## Abstract

**Objective:**

To determine the occurrence and characteristics of atelectasis, opacities, hypolucency and pulmonary infiltrates observed on chest X-rays of preterm infants in a neonatal intensive care unit.

**Methods:**

This was a cross-sectional observational study. From August to December 2017, all chest radiographs of newborn infants were analyzed. The study included the chest radiographs of preterm neonates with gestational ages up to 36 weeks in the neonatal period that showed clear changes or suspected changes, which were confirmed after a radiologist’s report. Radiological changes were associated with possible predisposing factors.

**Results:**

During the study period, 450 radiographs were performed on preterm neonates, and 37 lung changes were identified and classified into 4 types: 12 (2.66%) changes were described as opacities, 11 (2.44%) were described as atelectasis, 10 (2.22%) were described as pulmonary infiltrate, and 4 (0.88%) were described as hypolucency. A higher occurrence of atelectasis was noted in the right lung (81.8%). Among the abnormal radiographs, 25 (67.6%) newborn infants were receiving invasive mechanical ventilation.

**Conclusion:**

Considering the radiological report, no significance was found for the observed changes. Atelectasis was not the most frequently observed change. The predisposing factors for these changes were extreme prematurity, low weight, male sex, a poorly positioned endotracheal tube and the use of invasive mechanical ventilation.

## INTRODUCTION

Atelectasis is a condition that occurs due to the collapse of alveolar units. One of the main characteristics is a reduction in lung volume, which destabilizes the relationship between ventilation and perfusion and causes pulmonary shunting.^(1)^

Compared to the lungs of an adult, preterm neonates have different physiological and anatomical lung characteristics that predispose them to atelectasis. Due to lung immaturity, the number of alveoli is reduced, with low surfactant synthesis and the absence or underdevelopment of collateral ventilation, resulting in decreased lung compliance. In contrast, the chest cavity has increased compliance due to its cartilaginous structure.^(1-3)^

The main factors that contribute to atelectasis in premature neonates are the use of mechanical ventilation, poor endotracheal tube (ETT) placement, mucus plugs, elective or accidental extubation and diseases such as bacterial pneumonia, bronchopulmonary dysplasia, pleural effusion, respiratory distress syndrome, meconium aspiration syndrome, gastroesophageal reflux and pneumothorax.^(4)^

Typically, atelectasis is visualized on chest radiographs as increased density and decreased volume associated with decreased intercostal spaces, deviation of mediastinal structures, such as the trachea and the heart, elevation of the ipsilateral diaphragm and hyperinflation of the contralateral lung.^(5)^ However, technical problems, such as X-ray beam underpenetration and inadequate centralization, compromise the quality of the exam, which may result in nonspecific imaging features described as hypolucency or opacity.^(6,7)^

Another radiological feature frequently observed on the chest X-rays of premature neonates is hypolucency, which may suggest pulmonary edema, hemorrhage, atelectasis or consolidations. Opacities may occur due to lung collapse or pleural effusion and to pulmonary infiltrates attributable to the accumulation of fluid in the interstitial space as a result of some inflammatory process.^(6,8)^ However, for an accurate diagnosis, these radiological changes must be associated with other classic signs of the suspected disease.^(4)^

Specific radiological features may appear on a chest radiograph and may affect a lobe, a segment or the entire lung.^(9)^ Chest radiography is one of the most commonly used tests to show classic signs of atelectasis, opacities, hypolucency and pulmonary infiltrates. Chest X-ray is an important tool for diagnostic support and clinical evaluation of patients admitted to the intensive care unit (ICU).^(1)^

For physiotherapists working in this area, knowledge about the radiological findings specific to each change on chest images and their associated factors is of utmost importance because they provide guidance for determining the most appropriate plan for a patient. As a result, the present study aimed to assess the occurrence and characteristics of atelectasis, opacities, hypolucency and pulmonary infiltrates in preterm infants (PTIs) and to verify these features' locations and possible associated factors.

## METHODS

This is a prospective cross-sectional observational study conducted at a neonatal ICU of a public university hospital from August to December 2017. The project was approved by the Institutional Ethics Committee of the institution according to opinion 2.192.954.

Chest radiographs were performed following the medical referral routine in preterm neonates admitted to the hospital with suspected pulmonary alterations. Data were collected daily. Cases that met the inclusion criteria were selected: chest radiographs of PTIs with a gestational age (GA) up to 36 weeks + 6 days admitted to the neonatal ICU (NICU) during the neonatal period. New cases of lung changes were considered when resolution of the previous change was observed in at least one normal imaging test. Informed consent forms were signed by the parents. The exclusion criteria were radiographs without adequate framing or radiological changes.

**Figure 1:**
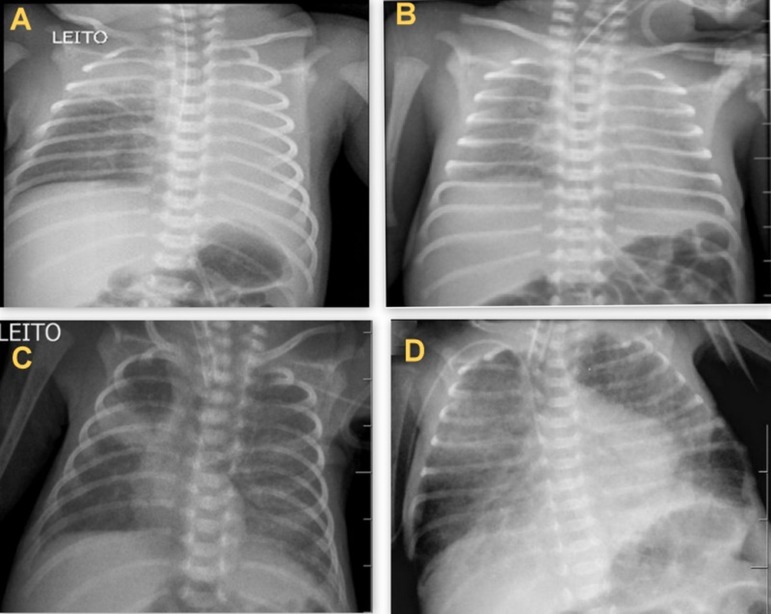
Radiological changes. (A) Atelectasis in the left lobe and right apex of the lung. (B) Diffuse hypolucency. (C) Opacity on the right middle third of the lung. (D) Diffuse pulmonary infiltrates.

Chest radiographs that showed lung changes (Figure 1) were selected and sent to a medical radiologist who analyzed the images and wrote a report blinded to the patient's clinical information to avoid inspection bias and ensure that only the imaging test contributed to the diagnosis.^(10)^ Then, data from medical records were collected, such as sex, GA, weight, 5-minute Apgar score, days of life, diagnosis and the use of some type of ventilatory support (invasive or noninvasive) or oxygen therapy.

The data were entered in a Microsoft Office Excel^®^ spreadsheet, reviewed and exported to Statiso Statsoft^®^ statistical software. Subsequently, the data were analyzed by descriptive statistics with the use of the absolute and relative frequencies, means, minimum and maximum values and standard deviation.

## RESULTS

**Figure 2:**
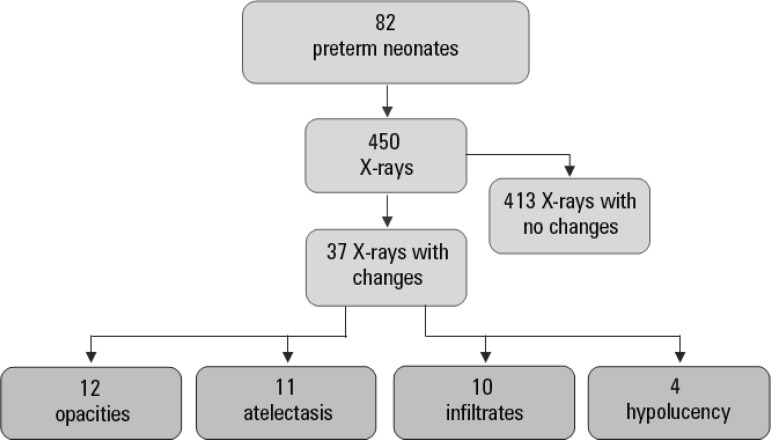
Flowchart of radiological changes in preterm neonates.

Between August and December 2017, 121 newborns were admitted to the NICU, 82 of whom were PTIs. A total of 713 chest radiographs were performed during this period, including 450 on PTIs, as shown in figure 2.

A total of 37 chest radiographs of the NICU patients were evaluated by the medical radiologist during the study. Of these, 12 (32.4%) lung changes were described as opacities, 11 (29.7%) were described as atelectasis, 10 (27%) were described as pulmonary infiltrates, and 4 (10.8%) were described as hypolucency. Considering only the 450 chest X-rays of the PTIs, 2.66% of the lung changes over four months were opacities, 2.44% were atelectasis, 2.22% were infiltrates and 0.88% were hypolucency.

The epidemiological characteristics of the sample and the clinical variables are shown in table 1. The clinical diagnoses of the preterm neonates were divided into two groups: pulmonary and extrapulmonary. Patients admitted due to respiratory distress syndrome and meconium aspiration syndrome were included in the group with pulmonary diseases. Patients admitted due to malformations and neonatal asphyxia were included in the extrapulmonary diagnosis group.

**Table 1:** Epidemiological characteristics of the sample and clinical variables

	Atelectasis	Hypo-small differences	Pulmonary infiltrates	Opacities
	11 (29.7%)	4 (10.9%)	10 (27%)	12 (32.4%)
Gestational age (weeks)	26.0 ± 3.4	27.3 ± 1.5	27.1 ± 1.8	29.6 ± 3.3
Up to 28	7	3	7	5
29 - 32	3	1	3	4
33 - 36	1	-	-	3
Weight (g)	1.390 (506 - 2.620)	1.120 (515 - 1.390)	637 (485 - 1.150)	931 (515 - 3.375)
Sex				
Female	3	-	1	7
Male	8	4	9	5
5-minute Apgar score				
8-10	2	2	2	3
3-7	7	1	-	3
0-2	2	1	8	6
Diagnosis				
Pulmonary	5	2	9	3
Extrapulmonary	6	2	1	9
Days of life	14 (1 - 29)	2,5 (1 - 4)	19 (2 - 24)	11 (1 - 28)
< 15	6	4	4	6

n = 37 preterm neonates. The results are expressed as the mean ± standard deviation; n or the median (minimum-maximum).

Nine patients exhibited recurrence of the different lung changes observed or even the same change at different periods, with at least one normal chest radiograph between the abnormal images. Two patients showed only one lung change on the chest radiograph during the neonatal period.

The types of ventilatory support that the PTIs received when they exhibited a change on chest X-rays are described in table 2. Of the 25 patients receiving invasive mechanical ventilation (IMV), 14 (56%) had a well-placed endotracheal tube (ETT). Of the 11 (44%) patients in whom the ETT was poorly positioned, 2 (8%) had an electively placed ETT positioned in the right primary bronchus, 3 (12%) had an ETT positioned high (above T1), and 6 (24%) had an ETT positioned low (close to the carina). Some type of lung change was noted in all the radiological exams in which the ETT was poorly positioned. The poorly positioned ETTs were adequately repositioned after chest radiography.

**Table 2:** Pulmonary radiological changes according to ventilatory support

	Atelectasis	Hypolucency	Pulmonary infiltrates	Opacities
n = 37	11 (29.7%)	4 (10.9%)	10 (27%)	12 (32.4%)
MV	7	2	9	7
NIV	1	2	-	3
NC	3	-	1	2

MV - mechanical ventilation; NIV - noninvasive ventilation; CN - nasal catheter. The results are expressed as n.

Lung changes were observed in two patients after accidental extubation, one of whom showed opacity while the other showed atelectasis. Two cases of atelectasis were caused by placement of the ETT in the right primary bronchus.

Nine cases of atelectasis were diagnosed in the right lung, one case was diagnosed in the left lung, and one case of bilateral atelectasis (with complete collapse of the left lung and collapse of the right apex) was diagnosed. Of the four X-ray examinations described as hypolucency, hypolucency was observed on the right side in two cases and affected both lungs in the other two cases. Of the ten cases described as pulmonary infiltrates, 8 had bilateral involvement, pulmonary infiltrates affected the middle third of the right lung in one case, and pulmonary infiltrates affected the base of the left lung in one case. Of the 12 cases described as opacities, 7 cases had opacities were on the right side and 5 had bilateral opacities. Regarding the side most frequently affected by the other lung changes, 19 (51.3%) changes were located in a lobe of the right lung, 2 (5.5%) changes affected some segment of the left lung, and 16 (43.2%) changes exhibited changes in some segment on the right and left sides (Table 3).

**Table 3:** Locations of pulmonary changes on chest radiographs

	Atelectasis	Hypolucency	Pulmonary infiltrates	Opacities
	11 (29.7%)	4 (10.8%)	10 (27%)	12 (32.4%)
Right apex	4	-	-	1
Right middle third	2	-	1	1
Right base	-	1	-	5
Total right	3	1	-	-
Left base	-	-	1	-
Total left	1	-	-	-
Bilateral	1	2	8	5

## DISCUSSION

Pulmonary structural immaturity associated with pulmonary surfactant deficiency often predisposes preterm neonates to respiratory function impairment with variable manifestations, such as lung changes.^(11)^ However, several associated factors may contribute to the development of these lung changes, such as the use of IMV, poor ETT placement, abdominal muscle weakness, accidental extubation and pulmonary complications during hospitalization.^(4,7)^

In the present study, 22 (59.4%) of the 37 preterm neonates with some lung change in the neonatal period were extremely premature infants with a GA of less than 28 weeks, which corroborates the study by Oliveira et al.^(12)^ confirming an association of GA with respiratory diseases and reporting that 50% of premature children developed respiratory complications.

The literature reports that low birth weight and GA are considered determining factors in the evolution of a newborn,^(12)^ which is similar to the results of the present sample because most (75.6%) of the preterm neonates weighed less than 1,500g.

Another factor that may have contributed to the appearance of lung changes was the use of IMV as 67.6% of the PTIs exhibiting lung changes on chest radiography were receiving IMV. A study characterizing the profile of preterm neonates in the NICU found that 93.8% of the children had respiratory diseases during hospitalization and 90.1% required IMV.^(12)^ This finding is relevant to the present study because respiratory diseases that lead to the need for IMV tend to evolve with the emergence of radiological changes on imaging tests.

Pediatric studies have shown that atelectasis is the most common complication in children undergoing mechanical ventilation.^(7,13,14)^ However, in those studies, atelectasis was compared with other clinical diagnoses or complications, such as accidental extubation, and not with changes on chest X-ray images.

In the chest radiographs included in the present study, 12 changes observed were described as opacities, representing the most common lung change. Atelectasis was described in 11 chest radiology images. The literature reports that when diagnosing atelectasis, an increase in local density (opacity) should be observed in the imaging test, indicating that the alveolar units may have collapsed. However, with opacity of a lung segment but no other associated features, atelectasis cannot be confirmed and hypotheses for alveolar collapse cannot be ruled out.^(1)^

Notably, the presence of opacity or atelectasis may correspond to alveolar collapse and loss of alveolar volume. Although most chest radiographs are not confirmed as atelectasis, specific treatments are needed due to signs of decreased aeration, lung volume and lucency on a chest image, such as physical therapy for pulmonary re-expansion, intermittent positive pressure breathing and noninvasive ventilation, to avoid invasive measures such as bronchoscopy and IMV.^(15)^

In a retrospective study evaluating atelectasis in patients under mechanical ventilation, 90% of the patients were found to have partial collapse of the right lung, confirming that the right side is more frequently affected due to the anatomical peculiarities of newborns.^(7)^

Two cases of elective ETT placement were noted, which caused atelectasis of the entire left lung in these PTIs. Elective intubation in newborns often results in ETT placement in the right primary bronchus due to its anatomical position being more straight than the left primary bronchus in addition to the difficulty associated with properly positioning an ETT due to the size of the airway.^(4,7)^

Lung changes may also occur after episodes of unscheduled extubation. In two patients, the lung changes observed on the chest X-rays after accidental extubation were opacity and atelectasis, which were caused by the abrupt loss of positive airway pressure, leading to pulmonary collapse. In a study evaluating the efficacy of a physical therapy protocol in patients after accidental extubation to prevent pulmonary collapse, approximately 20% of the newborns showed evidence of some degree of nonprogrammed postextubation collapse characterized by increased pulmonary opacity on radiographs within 24 hours after the event.^(16)^

The most common radiological abnormality found in patients receiving mechanical ventilation was pulmonary infiltrates. Sarmento et al.^(17)^ describe that in pulmonary diseases such as acute respiratory distress syndrome, meconium aspiration, pneumonia and bronchiolitis, this radiological change occurs due to an inflammatory process, which leads to collapse of alveolar units and a reduction in lung volume, in addition to causing an increased risk of infection associated with the use of mechanical ventilation.

Limitations of the present study were related to the small size of the sample, which was sampled by convenience. However, this small sample size is justified by the short period of data collection. Another limitation is related to the fact that X-ray is not a gold standard technique for diagnosing pulmonary atelectasis; therefore, the radiological reports suggest a change but do not confirm a diagnosis. Other studies are recommended with a greater number of PTIs and more specialist and nonspecialists examiners to compare the views of those who only evaluate images and those who assess the associations of the imaging features with the patient.

## CONCLUSION

Considering the radiological report, the observed changes showed no significant differences. Atelectasis was not the most frequently found change on the chest radiographs of preterm neonates; opacity was the most frequent finding. However, this finding does not rule out the hypothesis of lung collapse. The side most affected by atelectasis was the right side. Extreme preterm, low-weight male patients under invasive mechanical ventilation and with poorly positioned endotracheal tubes are more likely to develop lung changes that can be observed on chest radiographs. Recognition of the characteristics of each alteration leads to the most appropriate physiotherapeutic approach for each newborn infant, which may prevent the progression of more severe pulmonary complications.
